# How pre-trial qualitative research can change proposed RCT design: a case study and implications for future research

**DOI:** 10.1186/1745-6215-16-S2-O35

**Published:** 2015-11-16

**Authors:** Leila Rooshenas, Christel Mcmullen, Jonathan Mathers, Daisy Townsend, Jenny Donovan, Jane Blazeby

**Affiliations:** 1 University of Bristol, Bristol, UK; 2 University of Birmingham, Birmingham, UK; 3 Bristol Clinical Trials and Evaluation Unit, Bristol, UK

## Background

There is increasing integration of qualitative research within randomised controlled trials (RCTs). However, how these methods are applied at the pre-trial stage to inform trial design and conduct is less well explored. Here we describe how qualitative methods were used in a feasibility study to inform the design of a pilot RCT.

## Methods

Semi-structured interviews with purposely sampled clinicians (n=61) and patients (n=32) explored current clinical practice and views about the proposed trial interventions and protocol. Analysis used constant comparative approaches. Emerging findings were disseminated through focused reports/presentations to the study management group and steering committee as research progressed.

## Findings

Qualitative findings informed changes to fundamental aspects of trial design and proposed outcome measures. Clinicians' interpretations of the original interventions were variable, and descriptions of current practice/terminology consistently differed with assumptions made in the grant proposal and commissioned call for research. New outcomes were recognised, prompted by clinicians'/patients' priorities. Measuring and reporting these outcomes was deemed crucial for a full trial's potential to impact future practice. Interviews with clinicians also prompted new ‘in trial’ data collection requirements, as clinicians anticipated potential changes to their clinical practice to accommodate the trial interventions. These changes had potential to impact the primary outcomes, prompting recommendations to measure/record these practices during trial conduct.

## Conclusion

Qualitative research at the pre-trial stage can transform fundamental aspects of trial design/delivery. Presenting emergent findings as the research progresses is one approach to enhancing impact, facilitated by participation of qualitative researchers as core members of investigator teams.

